# Characterization and Pharmacological Evaluation of Anti-Cellulite Herbal Product(s) Encapsulated in 3D-Fabricated Polymeric Microneedles

**DOI:** 10.1038/s41598-020-63271-6

**Published:** 2020-04-14

**Authors:** Reham I. Amer, Ghada H. El-Osaily, Riham O. Bakr, Riham Salah El Dine, Ahmed M. Fayez

**Affiliations:** 1 Department of Pharmaceutics and Industrial Pharmacy, Faculty of Pharmacy, Al-Azhar University, Cairo, Egypt; 2 Department of Pharmaceutics, Faculty of Pharmacy, October University for Modern Sciences and Arts (MSA), Giza, Egypt; 3 Department of Pharmaceutics, Faculty of Pharmacy, Modern University for Technology and Information (MTI), Cairo, Egypt; 4 Department of Pharmacognosy, Faculty of Pharmacy, October University for Modern Sciences and Arts (MSA), Giza, Egypt; 5 Department of Pharmacognosy, Faculty of Pharmacy, Cairo University, Cairo, Egypt; 6 Department of Pharmacology, Faculty of Pharmacy, October University for Modern Sciences and Arts (MSA), Giza, Egypt

**Keywords:** Drug delivery, Quality of life

## Abstract

Skin health is vital for a healthy body. Herbal remedies have long been used for skin care, and their global use has tremendously increased over the past three decades. Although cellulite is seen as a normal condition by the medical community, it is considered a serious cosmetic concern for most affected women. Many topical anti-cellulite creams are available on the market, but unfortunately, their efficacy has not been proven scientifically. Microneedles (MNs) represent a new approach to enhance the permeation of loaded medication through the skin. In this study, the anti-cellulite effects of *Vitex agnus-castus* and *Tamarindus indica* extracts were compared using safe and effective polymeric MNs. This delivery system offers a painless alternative to the combined treatment strategy of microneedling devices and anti-cellulite products. The selected standardized extracts were evaluated for their mineral, phenolic and flavonoid contents, which are correlated to a promising antioxidant effect, as demonstrated by an *in vitro* radical scavenging activity assay. 3D-printing techniques were chosen for fabrication of a micromold, which is inexpensive for mass production. To ensure that MNs were sufficiently strong to perforate the skin without breaking, axial failure force was measured using a micro-mechanical test machine. The anticellulite effects of MNs were assessed using an *in vivo* diet-induced obesity guinea pig model. Skin properties, histopathology and inflammatory markers were examined. MNs loaded with plant extracts were statistically comparable in normalizing the oxidative state and reducing inflammation, while myeloperoxidase levels were more significantly reduced by *T. indica* than by *V. agnus-castus*. This novel delivery system opens the door for new transdermal strategies for cellulite management.

## Introduction

The skin is a chief barrier that provides protection for the human body. The outermost layer of the epidermis (stratum corneum, SC) is mainly responsible for this barrier property. Cellulite is a skin condition that affects up to 90% of women over 20 years of age and only 2% of men. It includes changes in skin appearance, with an orange-peel-like texture (mostly on the hips and buttocks) due to the expansion of fat lobules out of their connective frame and into the dermis^1–3^. Cellulite is a pathologically complicated condition that is associated with decreased microcirculation, oedema, overgrowth of adipocytes, oxidative stress, continuous inflammation, and changes in the extracellular matrix^4,5^. Some pharmaceutical products can treat cellulite by increasing the microvascular flow, inducing lipolysis, restoring dermis and connective tissue structures, and preventing alterations in the connective tissue produced by free radicals^6^. An effective delivery system for cellulite treatment should provide medication to the deep layers of the skin using painless, biodegradable, and consumer-friendly self-administration systems^7^. However, most active molecules are unable to penetrate the skin due to the skin’s barrier property. Low-molecular-weight molecules or those having log P (logarithm Partition coefficient) equal to 1–3 are characterized by their passive diffusion across the SC. Many innovations, such as iontophoresis, electroporation and acoustical methods, have been developed over the last decade to overcome this challenge^8^. Microneedles (MNs) are considered to be one of the most effective recently discovered hybrid techniques that have been developed to have the advantages of invasive (injection) and non-invasive (transdermal patches) drug administration routes. MNs are considered a promising technique for diagnostic, cosmetic, therapeutic and vaccination purposes^9–11^. MNs represent an advantageous transdermal delivery system (TDDS), as their needles act by penetrating the SC barrier and the upper dermal layers to deliver different molecules or to draw blood^12^. Drug transportation is better allowed by the formed microchannels in the SC, which disappear after a short time due to the body’s normal healing mechanism^9^. The selection of MN type and implementation technique is of considerable interest. The two main types of MN designs are solid or hollow ones. Stambini *et al*. fabricated two different types of hollow MNs: one was in the form of a silicon dioxide microchip, used for injection/sampling^13^, while the other was used as a transdermal biosensor, measuring glucose levels in the interstitial fluid with high accuracy and reproducibility^14^. Currently, MNs are used in transdermal biosensing of clinically important analytes^15^. Different materials have been used to fabricate MNs, such as metal, silicon and polymers. Polymeric MNs have mainly been used to solve the non-biodegradable and non-biocompatible problems of metal and silicon MNs. They provide suitable candidates for the rapid release of macromolecular medications. The polymers used may be swellable, dissolvable or biodegradable. Swellable MNs (e.g., polyvinyl alcohol) swell in the skin but do not dissolve, in contrast to dissolvable MNs (e.g., polysaccharides such as dextran, dextrin or sodium alginate), which completely dissolve after application^16,17^. Biodegradable MNs do not swell or dissolve in the skin; rather, they degrade, like those made of polylactic acid and chitosan^18,19^. Various methods are used to fabricate polymeric MNs, such as micro-moulding, drawing lithography, droplet-borne air drawing, photolithography, dipping^18^, and Particle Replication In Non-wetting Template (PRINT), which includes soft lithography in addition to conventional polymerization^20^. Micro-moulding is the most common method, as it is easy scalable and reproducible. This method requires fabrication of a master mould, casting polymeric solution in the master mould, ensuring complete filling with removal of air bubbles by centrifugation or vacuum, solidification by drying, then peeling MN arrays from the mould^18^. Stereolithography (SLA) is a type of 3D printing technique that is used for master mould fabrication to create a layer-by-layer structure in which a laser beam is concentrated to a photosensitive liquid-free surface to allow polymerization of liquid in that area and to convert it to a polymerized solid^21,22^. New strategies utilizing MN applications with topical anti-cellulite cream have been developed. ‘Dermaroller^®^, Germany, manufactured a device called the Beauty mouse. This combination helps in enhancing skin sensitivity to the anticellulite agent through the creation of microchannels in the skin^9^.

The use of herbal remedies has increased because of their therapeutic effects and the relatively few side effects of these medications compared to other current treatments^23^. *Tamarindus indica* L. (Fabaceae) is a fascinating plant with various applications in folk medicine and is used to treat rheumatism, cough and jaundice. *T. indica* (leaf, seeds and fruits) exhibited strong antioxidant^24–26^, anti-inflammatory^27^, hepatoprotective^28^ and hypolipidaemic^29^ effects. These activities are associated with high levels of phenolics, flavonoids^30^, fatty acid and mineral contents^31^. A water/oil emulsion formulation loaded with *T. indica* seed extract showed an effective anti-aging potential that was well correlated with a high antioxidant effect^32^. *Vitex agnus-castus* L. (Verbenaceae), or chaste tree, has a long history of being used to treat gastro-intestinal disorders, as a diuretic and as an anti-anxiety medication, in addition to the previously reported use of the fruits of *V. agnus-castus* in treating gynaecological disorders^33^. Many biological studies have shown *V. agnus-castus* to be a good candidate in herbal medicine, with antioxidant^34^, antimicrobial, anticancer and anti-inflammatory activities^35,36^, in addition to the hormonal effects of this plant leading to the incorporation of its berries in a formula called “Densorphin, Mibelle group, Switzerland” for improving skin elasticity^37,38^. Phytochemical screening of *V. agnus-castus* showed the presence of volatile oils, iridoids, and flavonoids in addition to phenolic acids^33,39–41^.

The present study aimed to test the potential of extracts of *V. agnus-castus* and *T. indica* leaves in the management of cellulite via different mechanisms of action upon encapsulating them within MNs that release the medication into the subcutaneous tissue with the dissolution of the needle itself, providing promising results in the improvement of the signs and symptoms of cellulite. The microneedle delivery system represents a novel tool that is safe, efficient and self-administered, without the need for suctioning the excess fat using high-cost ultrasonic liposuction, and decreasing the systemic side effects of oral slimming medications.

## Materials and methods

### Materials

Polyvinyl pyrrolidone K-30 (PVP K-30) was kindly provided by the Arab Drug Company for Pharmaceuticals and Chemical Industries (ADCO, Egypt). Chitosan (L.M.W.) was purchased from Sigma. Galactose and dextran (L.M.W) were kindly supplied by the Egyptian International Pharmaceuticals Industries Company (EIPICO). Sodium alginate was purchased from SISCO Research Laboratories (SRL). Spectrapore® nitrocellulose membranes (12,000 Da MW cut-off) were purchased from Spectrapore Inc. (New York, NY, USA). Polyvinyl alcohol (PVA), polyethylene glycol 400 (PEG 400) and ethanol were purchased from Al-Nasr Company for Chemicals and Pharmaceuticals, Cairo, Egypt. Gallic acid, quercetin, and ferulic acid were purchased from Sigma. Folin-Ciocalteu reagent, aluminium chloride, sodium bicarbonate, 2,2-diphenyl-1-picrylhydrazyl (DPPH), collagenase from *Clostridium histolyticum* (ChC, EC.3.4.23.3), methanol, and trifluoracetic acid (HPLC grade) were also used. Biocompatible Class 1 resin (Dental SG, by Formlabs, Germany) was used to fabricate the MN mould.

### Plant material and extraction

*T. indica* and *V. agnus-castus* leaves (0.5 kg, each) were collected from the Zoological Garden (Giza, Egypt) in October 2017. The leaves were kindly identified by Dr Threse Labib (Orman Botanical Garden in Giza city). A voucher sample (RS13) was deposited in the Herbarium of the Faculty of Pharmacy, October University for Modern Sciences and Arts (MSA). Dried and pulverized leaves were defatted with hexane and then extracted with 70% methanol under reflux (5 L × 5, 60 °C) until exhaustion. Finally, the aqueous methanolic extract was concentrated using a rotary evaporator to yield a viscous residue (40 g) that was lyophilized for further studies.

### Phytochemical study

#### Phenolic and flavonoid content

The aqueous methanolic extracts of *T. indica* and *V. agnus-castus* (0.1 g each) were dissolved in 25 mL of methanol, and 0.1 mL of each extract was then used to estimate the total phenolic (TP) content using the Folin-Ciocalteu method^42^. TP levels were expressed in terms of gallic acid equivalents (GAE) on the basis of a standard curve of gallic acid (10–100 µg/mL, Y = 8.097x, R^2^ = 0.985). The absorbance of the reaction mixture was recorded against a blank at 715 nm by using a UV spectrophotometer. Additionally, the total flavonoid (TF) content was estimated for the same extracts using the aluminium chloride assay as quercetin equivalents based on a quercetin calibration curve (25–200 µg/mL, Y = 0.002x, R^2^ = 0.985)^43^. The absorbance of the reaction mixture was recorded against a blank at 510 nm by using a UV spectrophotometer. All procedures were performed in triplicate.

#### Standardization of plant extracts

Preparation of standard and test solutions. Ferulic acid and 10 mg of the crude extracts were dissolved in 5 mL of methanol, shaken vigorously and centrifuged at 3000 rpm for 5 min. Then, the supernatants were filtered through a polyvinylidene difluoride filter (0.45 *μ*m). The ferulic acid calibration curve showed a linear regression of R^2^ = 0.991 for a concentration range of 5.6–16 μg/mL.

HPLC conditions: Analysis of *V. agnus-castus* and *T. indica* was conducted using an HPLC system (Agilent Technologies, Germany) that consisted of a separation module (2695), a photodiode array detector (2998), and Empower 2 data processing software (Waters, Milford, MA, USA). Chromatographic separation was carried out on a symmetry C_18_ column (100 mm × 4.6 mm i.d., Waters) maintained at 35 °C. The mobile phases were methanol (a) and 0.5% trifluoroacetic acid (b), with the following gradient programme: a/b = 5/95 (0 min)→30/70 (5 min)→90/10 (35 min)→100% (36–41 min) →5/95 (42–45 min), followed by holding for 5 min at 100% methanol at a flow rate of 1.0 mL/min. The detector collected all the spectral information between 210 nm and 450 nm. The injection volume was 20 *μ*L. Detection of ferulic acid was carried out at 280 nm on a diode array detector. The concentration of the compound in the samples was estimated based on the standard curve generated from a pure ferulic acid standard that was run under the same conditions as those described above.

#### Mineral content

Minerals with potential effects on skin health were assessed^44^. One gram each of *V. agnus-castus* and *T. indica* aqueous methanolic extracts was digested by wet digestion using concentrated sulphuric acid and a mixture of copper sulphate and anhydrous sulphate (1:10). The digested solutions were measured by using an atomic absorption spectrometer.

### 2,2-Diphenyl-1-picrylhydrazyl (DPPH) antioxidant assay

The free-radical scavenging activities of both aqueous methanolic extracts were measured with the DPPH assay, where both extracts were assayed at 25–75 µg/mL using a 0.1 mM methanolic solution of DPPH^•^, wherein the absorbance was measured at 517 nm using an Asys microplate reader compared with butylated hydroxyanisole (BHA) and vitamin C as a positive control^45^. The IC_50_ was determined; the DPPH scavenging effect (%) = 100 − [((A_0_ − A_1_)/A_0_) × 100], where A_0_ was the absorbance of the control reaction and A_1_ was the absorbance in the presence of the sample. The low IC_50_ values indicate high free-radical scavenging activity^46^. All the measurements were carried out in triplicate.

### Pharmaceutical formulation

#### Mould design and fabrication

**Figure 1:**
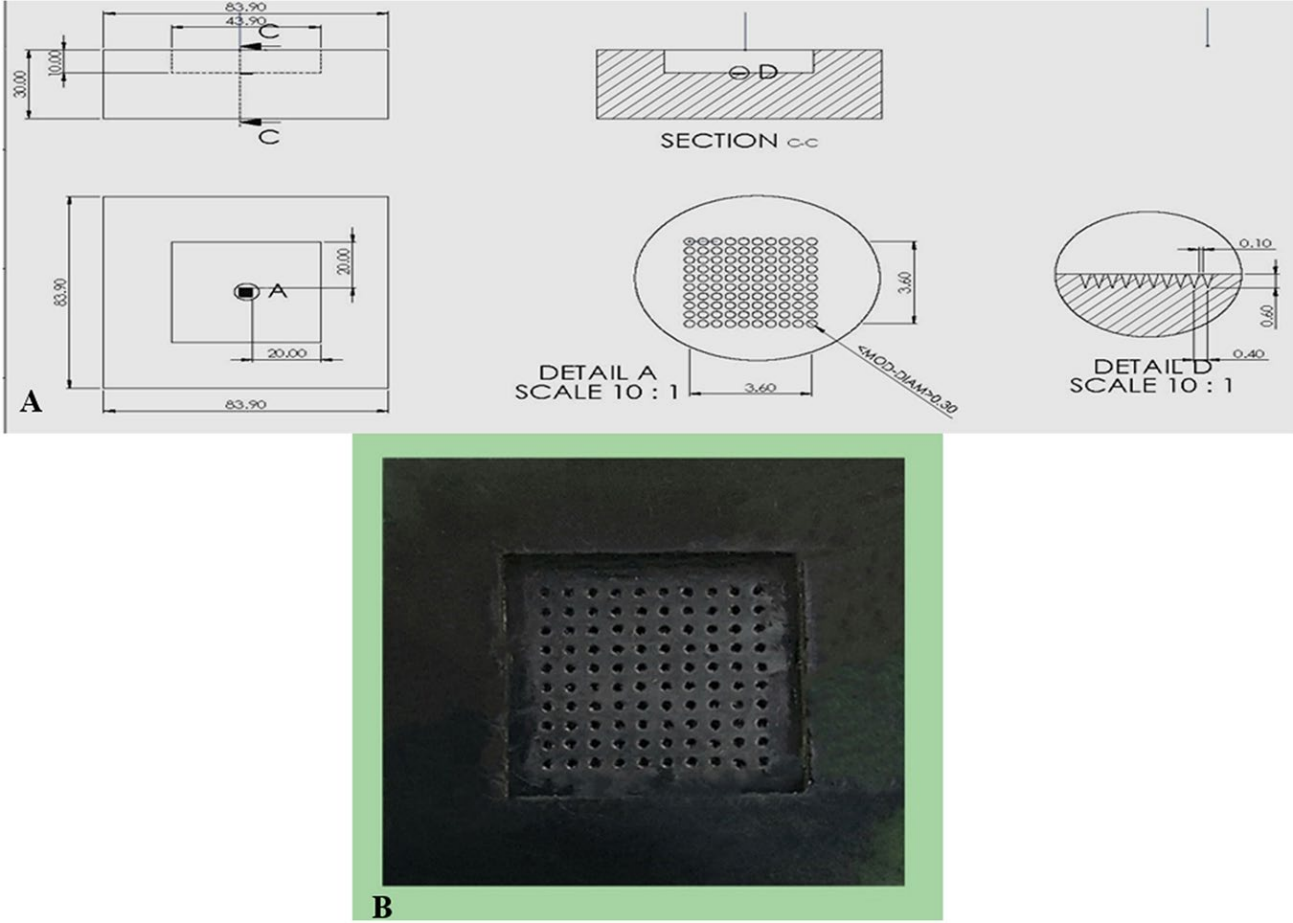
(**A**) Sketch diagram representing the geometrical shape and dimensions of the fabricated MNs mould, (**B**) Photograph of Stereolithographic fabricated MNs master mould.

A mould of a 10 × 10 array with length of 600 *μ*m, a base width of 300 *μ*m and an interspacing of 100 *μ*m was fabricated using stereolithography (SLA) and Computer-Aided Engineering (CAE) software files. CAE files are digitalized representations of conical MNs. Conical MNs are produced in MicroChem (SU-8 photoresist) via UV exposure^21,47,48^ (Fig. 1A). The process begins when the laser beam draws the first layer of the print into the photosensitive resin, and the liquid turns into solid. After performing the first layer, the platform is raised regarding the layer thickness, and more resin is permitted to flow under the already-printed portion. The laser then hardens the following cross-section, and the process is continued until the entire part is complete. Finally, it is exposed to UV light for extra hardening^22^. The fabricated mould was then used to produce the master structure of the MNs using different polymers (Fig. 1B).

#### Formulation of non-medicated polymeric microneedles (MNs)

**Figure 2:**
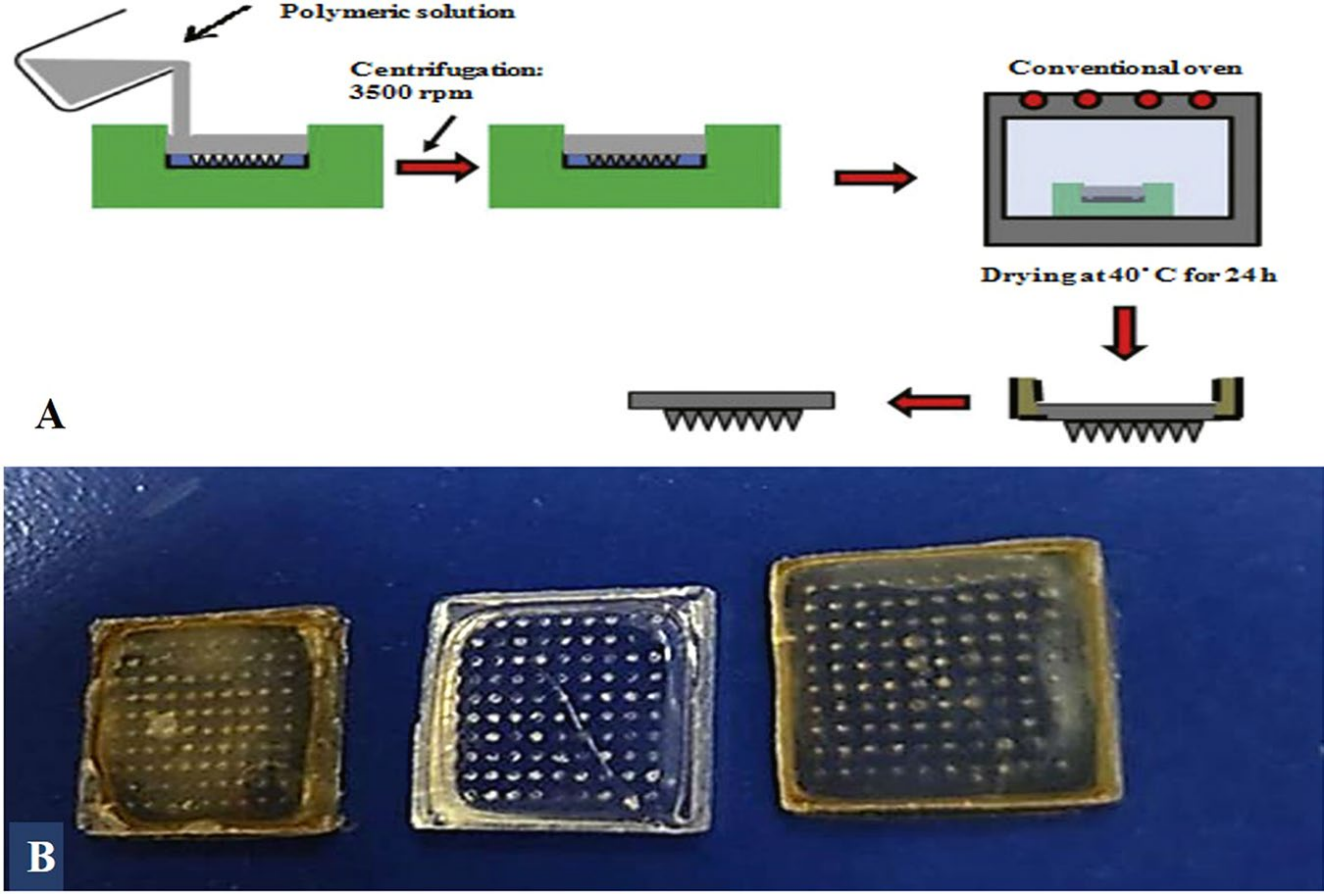
(**A**) Diagrammatic representation of different steps involved in polymeric MNs formulation^50^ (**B**) photograph of different 10 × 10 polymeric MN arrays.

Six polymers with different concentrations (Table 1) were used to fabricate non-medicated polymeric MNs of galactose or sodium alginate with an optimum consistency. The required amount of each polymer was dissolved in distilled water, while 1% glacial acetic acid was used as a solvent to prepare the chitosan polymeric solution. One hundred milligrams of each polymer solution gel was injected into the fabricated mould using a 1-mL sterile disposable needle-free syringe. The moulds containing samples were centrifuged at 3500 rpm for 45 min to remove air bubbles and to ensure the complete filling of pores and were then left to dry in the oven at 40 °C for 24 h^49,50^. Finally, the MN arrays formed (Fig. 2) were carefully withdrawn from the mould.

**Table 1:** Formulation of non-medicated polymeric MNs arrays.

Formulation	Polymers used	Polymer concentration (%w/w)
MN1	Chitosan	3
MN2	PVA	15
MN3	PVA:PVP K_30_ (1:1)	20
MN4	Dextran	20
MN5	Galactose	5
MN6	Sodium alginate	10

#### Preliminary evaluation of non-medicated MN arrays

Mechanical properties of polymeric MNs. MN skin insertion test. The insertion of MNs through the skin was examined on the surface of hairless guinea pig skin after elimination of subcutaneous fat. The skin was glued under slight pressure to a wooden plate utilizing 1-cm-long screws. Then, the insertion of fabricated MNs against the guinea pig skin was helped by placing a known weight (2 g) over it for approximately 10 min^51^. The site of array insertion on the surface of the skin was treated with a red tissue-marking dye (Safranil) for 10 min to stain the perforation formed in the SC. The skin was examined by light microscopy after removal of the remaining dye from the skin using dry tissue paper.

#### Mechanical failure force measurement

**Figure 3:**
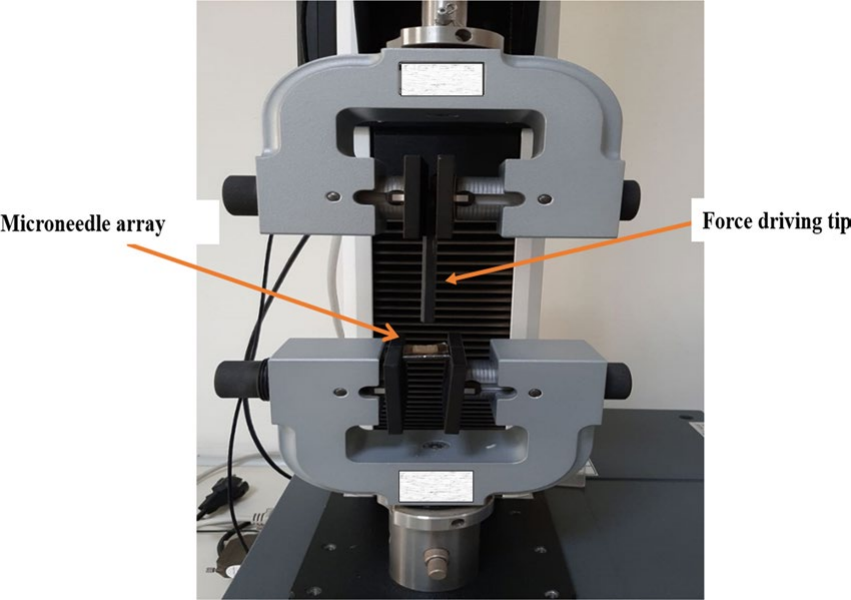
Optical image mechanical set up for application of compressional force to MN array.

All non-medicated polymeric MNs were subjected to a mechanical failure test using a micro-mechanical test machine (Instron®, model 3345, USA) (Fig. 3). The axial load that reflects the force applied parallel to the MN array axis was measured to determine the ability of polymeric MNs to withstand the force before failure under this load. The MN array was connected to the mount of a moving sensor, and an axial force was used to move the mount at a speed of 500 mm/min. The mount pushed the MNs against a hard metal surface that was perpendicular to the axis of mount movement. Upon needle failure, the force unexpectedly declined; the ultimate force applied instantly before this decline was recorded as the force of MN failure^52,53^. Data are represented in Table 2 as the mean values (n = 3 ± SD). Statistical analysis was carried out to compare the obtained results using one-way analysis of variance (ANOVA) followed by Tukey’s Multiple Comparison *post hoc* test; P ≤ 0.05.

**Table 2:** Mechanical Axial failure force measurements of different non-medicated polymeric MNs.

Formulations	Axial failure force/MN array (N) ± SD	Compressive stress at break (MPa) ±SD
MN1	6.432 ± 0.4717	0.6432 ± 0.04258
MN2	4.883^a^ ± 0.1258	0.4883 ± 0.02281
MN3	4.017^a^ ± 0.0764	0.4017 ± 0.0156
MN4	5.922^bc^ ± 0.4015	0.5922 ± 0.0411
MN5	3.755^abd^ ± 0.3606	0.3755 ± 0.04080
MN6	7.438^abcde^ ± 0.4325	0.7438 ± 0.0262

Data are presented as (mean ± SD, n = 3).

Statistical analysis was carried out by one-way analysis of variance (ANOVA) followed by Tukey’s Multiple Comparison *post hoc* test; P ≤ 0.05. As compared to (a) MN1, (b) MN2, (c) MN3, (d) MN4, (e) MN5.

#### Morphological characterization of MN arrays

 A cross-section of sodium alginate MN arrays was mounted on a circular disc and characterized morphologically using scanning electron microscopy (SEM, high-vacuum mode, 15 kV). The MN sample was first gold-coated using a sputter coater and dried using an ion beam-based system containing a single vacuum; then, computer software was used for imaging by SEM^54^. For additional morphological investigations, a light microscope was used to examine a portion of the 10 × 10 MN arrays.

#### Formulation of medicated MN arrays

Dried extracts in the lyophilized form of both *V. agnus-castus* and *T. indica* at two different concentrations were loaded in 10 mg of sodium alginate and then mixed with 100 mL of water to produce four medicated formulations. F1 and F2 contained *V. agnus-castus* lyophilized extracts in amounts equal to 100 and 200 mg, respectively, while F3 and F4 contained *T. indica* lyophilized extracts in amounts equal to 100 and 200 mg, respectively. The medicated polymeric solutions were poured into the mould cavities. Centrifugation was carried out at 3500 rpm for 45 min to ensure complete filling of the micro-cavities, after which the moulds were left to dry in the oven at 40 °C for 24 h. Addition of powdered extract to the non-medicated MNs increased the hardness and brittleness of the MNs upon removal from the mould; 1 mL of PEG 400 was added to overcome this difficulty. Finally, the produced arrays contained 100 needles perpendicular to the conical base.

#### Percentage of drug content

To determine the actual amounts of both extracts loaded into the prepared MNs, the arrays of different formulations were soaked in 3 mL of phosphate-buffered saline (PBS, pH 7.4) until dissolution was complete. The drug content was then determined spectrophotometrically, with absorption maxima at 340 nm and 278 nm for *V. agnus-castus* and *T. indica*, respectively. Samples were run in triplicate, and the mean values (n = 3 ± SD) are tabulated in Table 3.

**Table 3:** Percentage drug content and *in-vitro* drug release.

Formulations	Drug content (%) ± SD	% drug release after 90 min.
F1	95.01 ± 1.90	87.95 ± 1.4
F2	97.90 ± 1.25	96.90 ± 2.6^a^
F3	96.20 ± 1.20	90.4 ± 3.4^b^
F4	98.95 ± 2.10	98.01 ± 1.6^a,c^

Data are presented as (mean ± SD, n = 3).

Statistical analysis was carried out by one-way analysis of variance (ANOVA) followed by Tukey’s Multiple Comparison *post hoc* test; P ≤ 0.05. As compared to (a) F1, (b) F2, (c) F3.

#### In vitro drug release studies

*In vitro* release studies of *V. agnus-castus* and *T. indica* extracts from different polymeric MN formulations (F1-F4) were performed using a Franz diffusion cell^54^. Spectrapore® nitrocellulose membranes were soaked overnight in PBS (pH 7.4). Then, the medicated MNs were punctured into the pre-soaked membranes and loaded into the diffusion chamber using the pressure of a cylindrical stainless steel (4 g) weight on top of the MN arrays for approximately 5 s, after which the weight was removed. The receiver compartment containing PBS (pH 7.4) was degassed before use and thermostated to 37 ± 1 °C. Then, the donor compartment of the diffusion cell was clamped onto the receiver compartment. Three hundred microliters of each sample was removed from the Franz cell at different time intervals and replaced with pre-warmed PBS. Samples were filtered using filter paper discs (0.45 µm), and the amounts of *V. agnus-castus* and *T. indica* extracts released were determined using a UV spectrophotometer. Data are represented in Table 3 as the mean values (n = 3 ± SD). Finally; statistical analyses were carried out to compare the *in vitro* permeations of both extracts from different polymeric MNs after 90 min using one-way analysis of variance (ANOVA) followed by Tukey’s Multiple Comparison *post hoc* test; P ≤ 0.05. All collected data were then fitted to different kinetic models.

#### Kinetics study

To study the *in vitro* drug release mechanisms of both natural extracts from different MN formulations, the *in vitro* release data were fitted to the general exponential function M_t_/M_∞_ = kt^n^, where M_t_/M_∞_ represents the fractional uptake of solvent (or release of solute) normalized to the equilibrium conditions; n is a diffusion exponent characteristic of the release mechanism; and k denotes properties of the polymer and the drug. This equation describes the relative significance of Fickian (n ≤ 0.5) and Case II (n ≤ 1.0) transport in anomalous diffusion. Kinetic studies were performed by adjusting the release profiles to Higuchi, first-order and zero-order kinetics equations^55^.

### Pharmacological study

#### Experimental animals and study groups

A total of 24 female guinea pigs, weighing 185–200 g, were purchased from Ghazaly Animal Supplier, Cairo, Egypt. The animals were kept in the animal house of the Faculty of Pharmacy, October University for Modern Sciences and Arts (MSA), Egypt, under suitable humidity and temperature conditions (humidity 60–70%, temperature 24 ± 2 °C). The animals were fed standard pellet chow (El-Nasr Chemical Co., Cairo, Egypt) and were given water *ad libitum* in addition to lettuce and carrot. All experiments were performed in accordance with the guidelines of the Ethics Committee for Animal Experimentation at the Faculty of Pharmacy, MSA University and they were approved by the previously mentioned committee with reference number PH1/EC1/2018PD. High-fructose corn syrup (HFCS, 55%) was purchased from the National Company for Maize Products (NCMP). The animals were fed HFCS for 60 days implementing a diet-induced obesity model (DIO)^56^. *V. agnus-castus* and *T. indica* (200 mg/kg) extracts loaded on MNs were administered to the tested guinea pigs. All other chemicals used were of analytical grade.

Four-week-old guinea pigs were divided into four groups of six guinea pigs each. The normal control group (group 1) received standard chow for 60 days; group 2 was orally administered HFCS (55% w/v) twice daily for 60 days; group 3 was orally administered HFCS twice daily for 60 days and then treated with *V. agnus-castus* (200 mg/kg) loaded on MNs for 14 days; and group 4 was orally administered HFCS twice daily for 60 days, followed by treatment with *T. indica* (200 mg/kg) loaded on MNs.

To assess the effect on cellulite, body weight was recorded every 10 days. On day seventy-five, blood was collected from each animal aseptically by using sterile, disposable 3-mL syringes via cardiac puncture and was then centrifuged at 3000 rpm for 15 min. Serum was separated and stored at −20 °C for the detection of different biomarkers. Skin was excised, rinsed in ice-cold saline and fixed in 10% formalin for histopathological examination for determination of skin elasticity and health with a digital microscope and from digital photomicrographs.

#### Assessment of biochemical markers for antioxidant activity

The levels of the oxidative stress marker reduced glutathione (GSH), a natural antioxidant in the body, were determined by a method based on the reduction of 5,5′-dithiobis-2-nitrobenzoic acid (DTNB) with monitoring at 412 nm using a commercial kit (Biodiagnostic, Egypt). The levels of malondialdehyde (MDA), an oxidative stress marker that is the end product of lipid peroxidation, were evaluated using a commercial kit (Biodiagnostic, Cairo, Egypt).

#### Assessment of inflammatory mediators

Adiponectin hormone and endothelial nitric oxide synthase (eNOS) levels were measured to determine the main causes of cellulite, and the levels of inflammatory markers such as tumour necrosis factor alpha (TNF-α) and myeloperoxidase (MPO) were measured using commercial kits (MyBioSource Inc., USA).

#### Histopathological study

Tissues were fixed in 10% formalin and embedded in paraffin, and the obtained tissue sections were then collected on glass slides, deparaffinized and stained with haematoxylin and eosin, and finally examined using a light microscope.

### Statistical analysis

Statistical analysis was carried out by one-way analysis of variance (ANOVA) followed by Tukey’s test for multiple comparisons.

## Results and Discussion

### Phytochemical study

Both *T. indica* and *V. agnus-castus* showed high phenolic content using the Folin-Ciocalteu method, with higher values observed for *T. indica* than for *V. agnus-castus* (123 ± 0.28 and 115*±0.37 mg/g GAE, respectively; *significant difference from *T. indica*). Statistical analysis was carried out by unpaired t test, P < 0.05, n = 3. Moreover, the total flavonoid content estimated using the aluminium chloride assay and calculated as quercetin equivalent showed a higher flavonoid content in *T. indica* than in *V. agnus-castus* (26.300 ± 0.63 and 16.670*±0.54 mg/g QE, respectively; *significant difference from *T. indica*). Statistical analysis was carried out by unpaired t test, P < 0.05, n = 3.

#### Several minerals play vital roles in skin health

The main minerals detected in both plants are presented in Table 4. Notably, the identified micronutrients suggest protection from photodamage by antioxidant activity, for which selenium represents a major component in *T. indica* and *V. agnus-castus* extracts (2.260 ± 0.12 and 2.260 ± 0.15 mg/kg, respectively), in addition to zinc (26.200 ± 0.13 and 29.190 ± 0.13 mg/kg, respectively), as well as copper (21.900 ± 0.23 and 27.74 ± 0.21 mg/kg, respectively), which is essential as an antioxidant and aids in collagen synthesis^57^.

**Table 4:** Mineral content of *T.indica* and *V.agnus-castus*.

		*T.indica*	*V.agnus-castus*
mg/kg	Se	2.26 ± 0.12	2.26 ± 0.15
	Cu	21.90 ± 0.23	27.74 ± 0.21
	Cr	<0.2 ± 0.05	52.74 ± 0.17
	Zn	26.20 ± 0.13	29.19 ± 0.13
	Mn	25.3 ± 0.24	84.52 ± 0.2
	Fe	239.1 ± 0.31	1651.45 ± 0.3
%	Mg	0.03 ± 0.001	0.27 ± 0.01
	P	0.11 ± 0.01	0.11 ± 0.01
	Ca	0.07 ± 0.002	0.2 ± 0.003
	K	0.11 ± 0.005	1.37 ± 0.01

**Figure 4:**
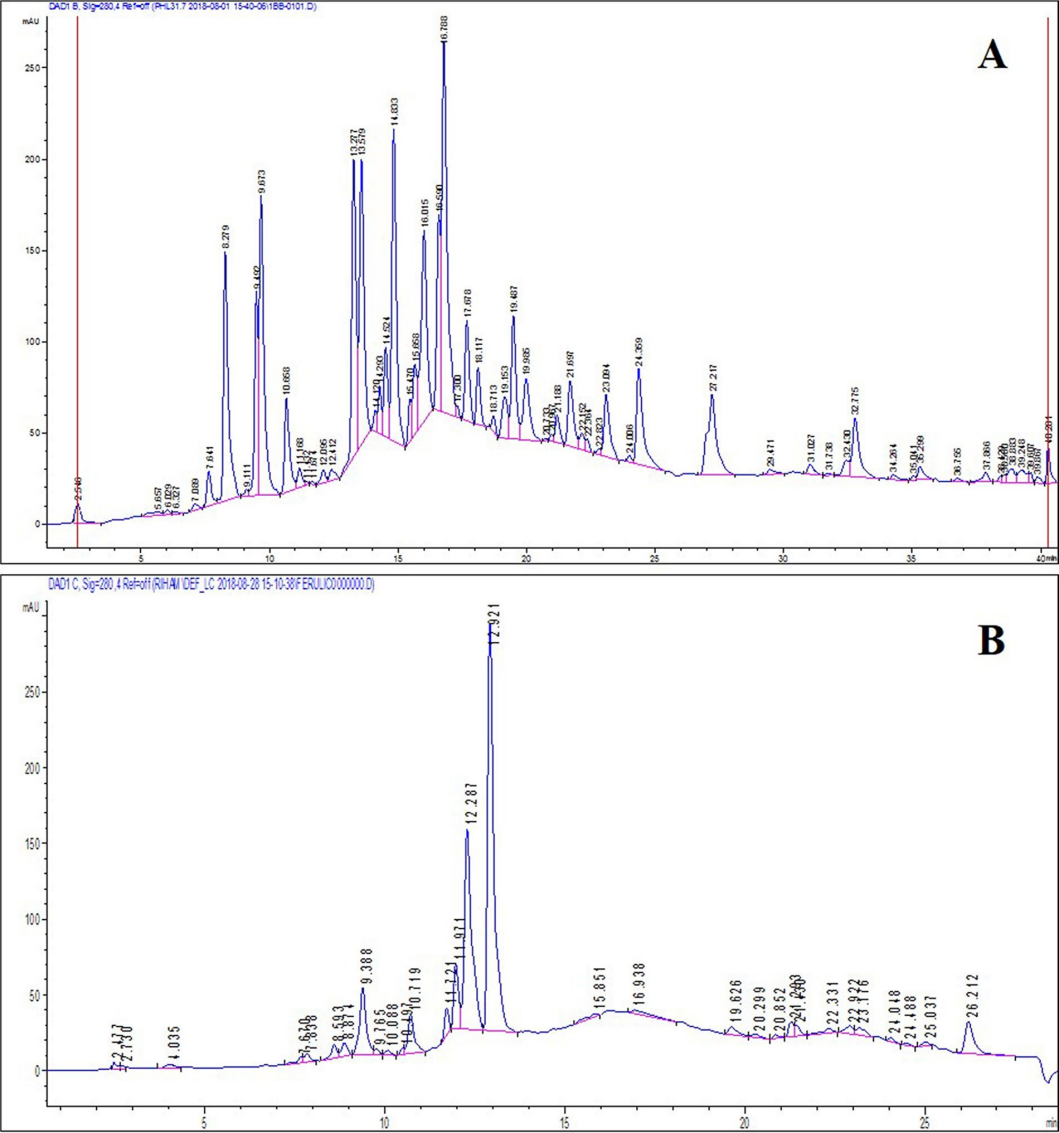
HPLC chromatogram of the aqueous methanolic extract of (**A**) *V.agnus-castus* and (**B**) *T.indica*.

**Figure 5:**
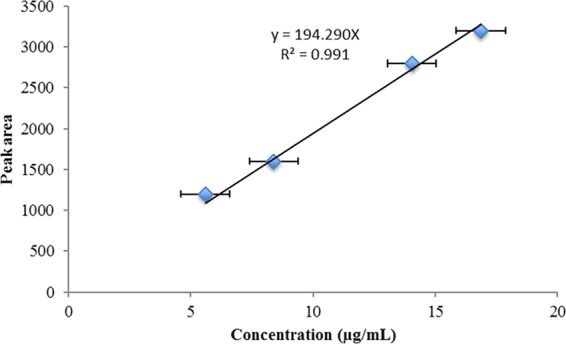
Standard calibration curve of ferulic acid.

The chromatograms obtained from HPLC analyses of the aqueous methanolic extracts of *T. indica* and *V. agnus-castus* showed the polyphenolic profiles of the two extracts (Fig. 4A,B). Ferulic acid was detected in both chromatograms at 280 nm based on comparison with the retention time of the ferulic acid standard and on the absorption spectrum of the standard obtained from the diode array detector. The ferulic acid response was linear in the range 5.6–16.86 *µ*g/mL, with a correlation coefficient of 0.991 (Fig. 5). The ferulic acid concentrations in the *V. agnus-castus* and *T. indica* leaf extracts were found to be 14.360 and 9.412 *µ*g/mL, respectively.

#### In vitro antioxidant activity

Free-radical scavengers are indispensable for the maintenance of healthy skin, delaying wrinkle formation and contributing to anti-inflammatory activity. The DPPH assay was used to test the antioxidant activity of the aqueous methanolic extracts of *T. indica* and *V. agnus-castus*. Consistent with the phytochemical study, a high free-radical scavenging activity was observed for *T. indica*, which exhibited low IC_50_ and IC_90_ values (15.8 ± 1.74 and 45.3 ± 0.65, respectively) compared with *V. agnus-castus*, which showed IC_50_ and IC_90_ values of 49.9^a^ ± 1.2 and 175.8^b^ ± 1.89* µ*g/mL, respectively (^a^ significant difference from *T. indica* IC_50,_^b^ significant difference from *T. indica* IC_90_). Statistical analysis was carried out by unpaired t-test, P < 0.05, n = 3. The values were comparable to those for vitamin C and BHA (12 ± 3.5 and 53 ± 3.1 *µ*g/mL).

### Pharmaceutical formulation

To develop a minimally invasive system to deliver anti-cellulite herbal medications across the skin and to offer the opportunity for continuous delivery, biocompatible polymeric MN arrays were selected and fabricated using the SLA technique. SLA is one of the common types of 3D-printing technology, in which superfine layering of a safe liquid resin is performed to construct a computer-designed shape. The liquid resin is contained in a reservoir, and laser energy is utilized to make individual layers that can be arranged to form shapes. These polymeric MN arrays were predicted to be mechanically strong due to their moderately high Young’s modulus^58^.

#### Mechanical properties of polymeric MNs

**Figure 6:**
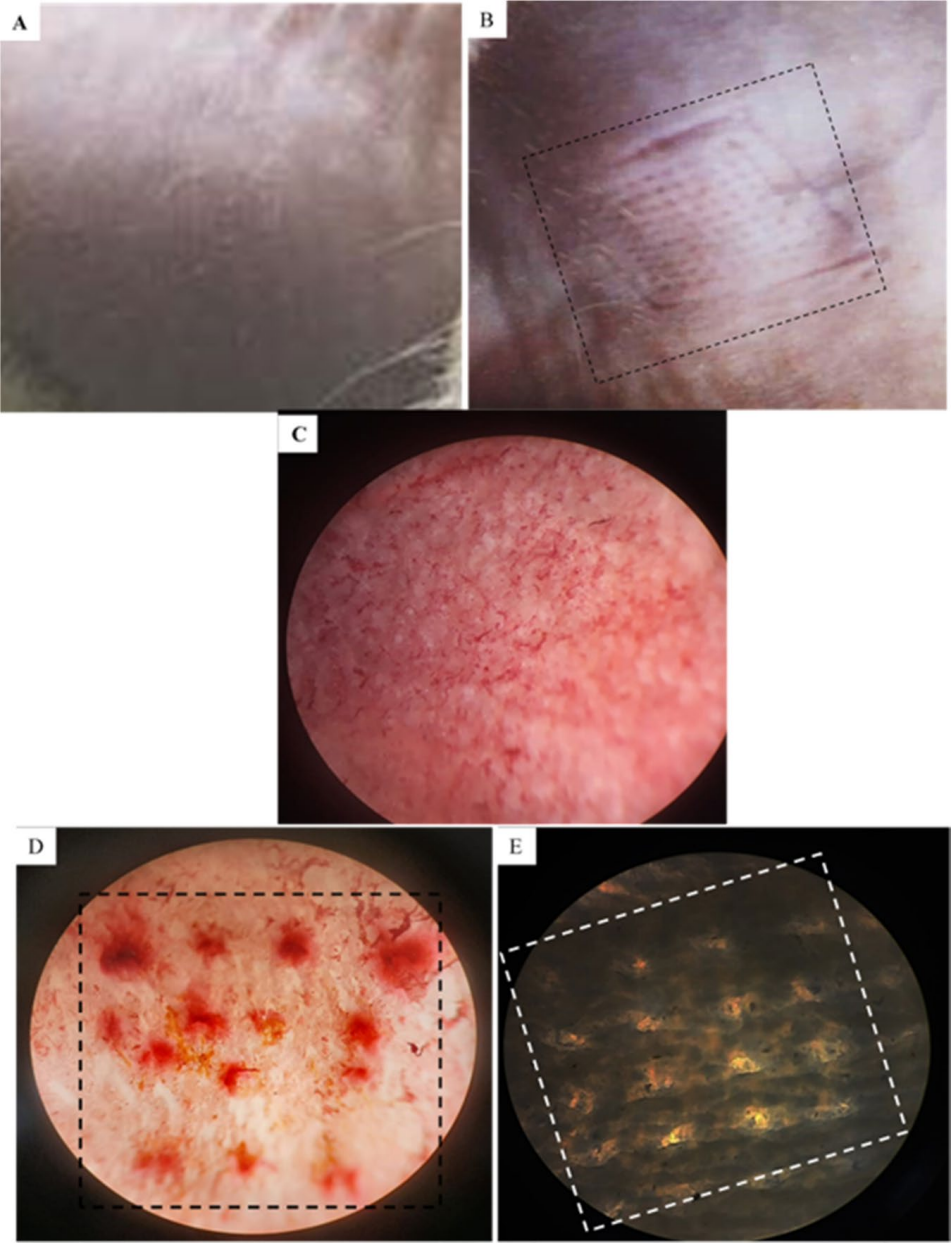
Surface of hairless guinea pig skin (**A**) before MNs insertion, (**B**) after insertion and removal of MNs, Photographs by light microscope for: (**C**) Guinea pig skin before MNs insertion, (**D**,**E**) Guinea pig skin after MNs insertion and removal with and without stain, respectively. Each stained spot reflecting the site of MN penetration into the skin.

**Figure 7:**
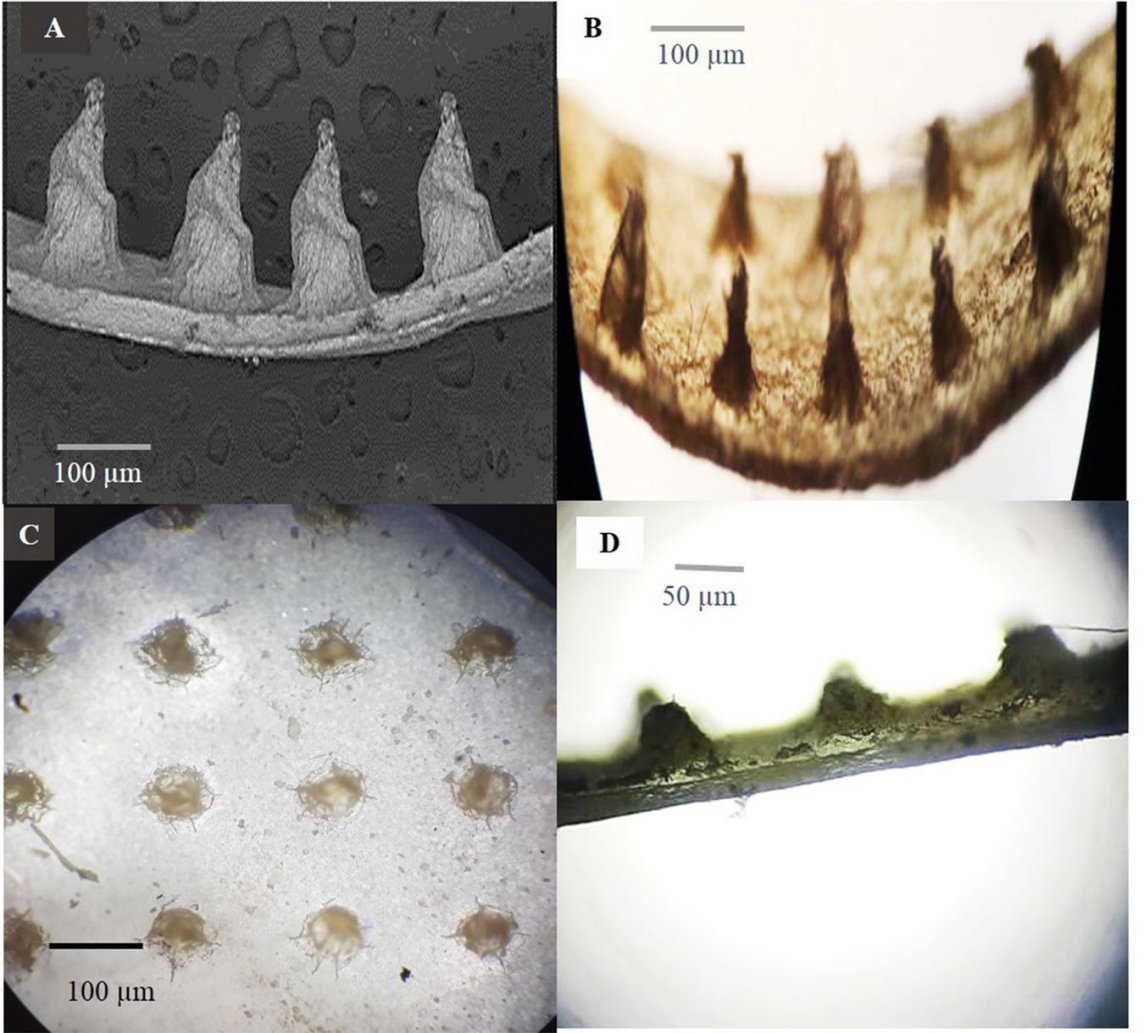
Cross sectional images of 10 × 10 MN arrays form: (**A**) Side view image by SEM, (**B**) Side view; (**C**) Top view; **(D**) Side view after insertion into the skin for 15 min as an evidence for complete dissolving of MN tips. Images (**B–D**) viewed by light microscope.

MN skin insertion test: The mechanical performances of different non-medicated polymeric MN arrays were investigated. By comparing the visualized results, a sodium alginate polymeric MN array (MN_6_) was selected as an optimized non-medicated polymeric MN array due to its ability to penetrate the skin easily. Additionally, this array produced highly visible micro-pores on the surface of guinea pig skin without any breakage (Fig. 6). The tips of the arrays began to directly dissolve, indicating the onset of rapid dissolution in the skin (Fig. 7D). The obtained results were consistent with Demir *et al*.’s observation comparing the insertion abilities of both PLGA-MNs and sodium alginate-MNs; it was noted that although biodegradable polymeric PLGA MNs exhibit relatively high durability and mechanical stability, the soluble sodium alginate MNs can make micro-perforations in the skin layers without fracturing^53^.

#### Morphological characterization of medicated MN arrays

The structure morphology of sodium alginate polymeric MNs was analysed by SEM (Fig. 7A). The obtained measurements confirmed the actual geometrical dimension of the MN master micro-mould, with viewed needle length (600–650 *µ*m), base width (300–350 *µ*m) and interspacing (100–125 *µ*m). Slight microneedle deformation was observed (i.e., needles become wider and more flattened); this modification in shape is attributed to the usage of low electron beam currents and a low magnification level during SEM sample preparation, which resulted in interaction between the tested sample and the electron beam, causing sample melting or even degradation^59^. The light microscopy photograph of the cross-section of a portion of the same MN sample obtained by vertical cutting (Fig. 7B) proved the presence of needles with uniform sharp tips, while (Fig. 7C) also confirmed the interspacing’s dimension between MNs.

#### Mechanical failure force measurement

To ensure that the prepared polymeric MNs were sufficiently strong to perforate the skin without breaking, the axial failure force was investigated using a micro-mechanical test machine (Instron^®^, model 3345, USA). The axial failure force of the different non-medicated MNs (Table 2) showed that as the molecular weight of the polymer increased, its mechanical strength increased, in agreement with the expectation and the conclusion of Mott^60^. It was also noted that blending of PVA with PVP in MN3 causes some decrease in its mechanical strength compared with the mechanical strength of PVA alone in MN2. The results were in agreement with previous findings demonstrating that as the ratio of PVP in its mixture with PVA increased, the mechanical strength decreased as a result of a faint interaction between PVA and PVP^18^. Statistical analysis confirmed that the axial failure forces of different non-medicated polymeric MNs were significantly different at P ≤ 0.05.

#### Percentage of drug content

The percentages of both herbal extracts in the different medicated MN formulations were calculated and are shown in Table 3. The percentage of drug content increased with increasing concentration of the herbal extracts in the examined MNs.

#### *In vitro* drug release studies

**Figure 8:**
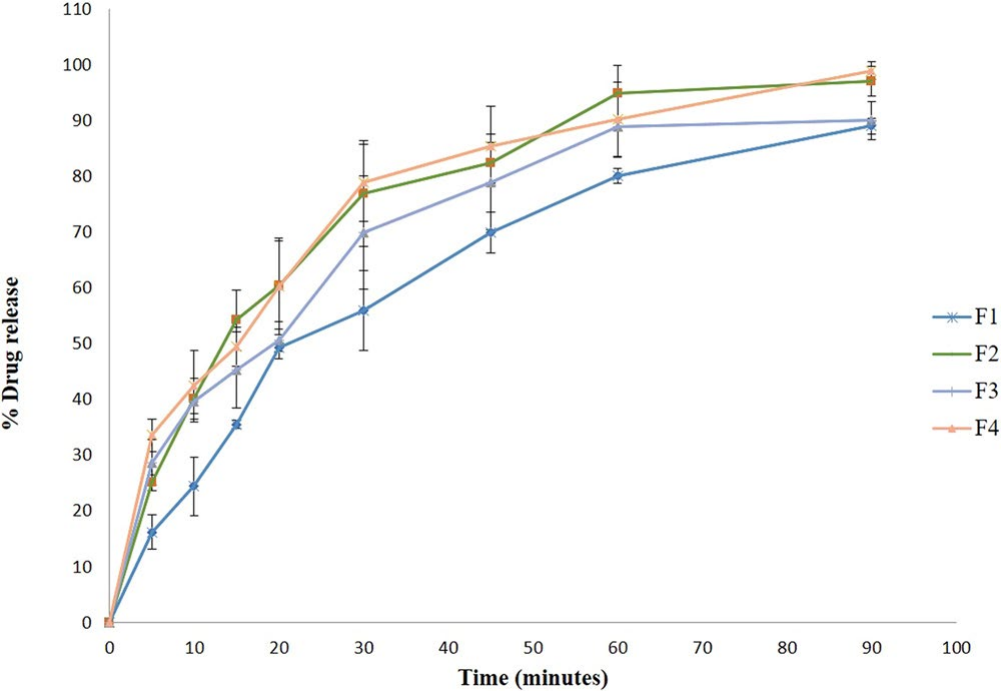
*In-vitro* drug release study of different medicated polymeric MNs formulations.

To estimate the characteristics of the drug release from the prepared polymeric MNs, an *in vitro* permeation study of all the medicated MN formulations was carried out. PBS buffer (pH 7.4) was used as a release medium, as it closely resembles the extracellular fluids and plasma. More than 90% of the medication encapsulated in the MN arrays was released within 90 min (Fig. 8). When the MNs were inserted into the skin, the dissolvable polymeric MNs started to quickly and completely dissolve in the skin without the generation of sharp biohazardous waste. The rapid dissolution of the MNs was accompanied by a rapid release of the encapsulated drugs, consistent with previous reports^61^. Finally, as the amount of the encapsulated extract in the prepared MNs increased, the amount of extract that permeated through the cellulose membrane increased. The maximum permeation of both extracts from the medicated polymeric MN formulations was observed in F2 and F4, containing 200 mg of *V. agnus-castus* and *T. indica*, respectively; therefore, they were considered for the *in vivo* study (Table 3), while the permeation from the other MN formulations was relatively low. Statistical analysis using ANOVA revealed that the permeation of both extracts from F2 and F4 after 90 min were significantly different at P ≤ 0.05.

#### Kinetics study

To understand the mechanism of drug release from different medicated MN formulations, the *in vitro* release data were fitted to Korsmeyer-Peppa’s release model; interpretation of the release exponent values (n) can help us understand the release mechanism of the MN arrays. As shown in Table 5, the values of the release exponents were 0.3501 to 0.7358. Based on these data, the F1 and F2 MN formulations exhibited Case II transport^61^, while the F3 and F4 MN formulations showed Fickian release. All the MN formulations showed high (r) values for first-order plots, indicating that the drug release followed first-order kinetics.

**Table 5:** Kinetic Profile of different medicated polymeric MNs formulations.

Formulation	Zero-order		First-order		Higushi-diffusion		Korsemeyer–Peppas			Possible kinetics order & mechanism of the drug release
	r	k	R	k	r	k	r	k	n	
F1	0.9546	0.8869	0.9959	0.0283	0.9903	10.908	0.9889	0.0485	0.7358	First order kinetics, Case II transport.
F2	0.8969	0.7981	0.9828	0.0392	0.9603	10.129	0.9997	0.0812	0.6984	First order kinetics, Case II transport
F3	0.9345	0.7765	0.9911	0.0295	0.9788	9.641	0.9971	0.1505	0.4076	First order kinetics, Fickian release
F4	0.9146	0.7568	0.9931	0.0404	0.9678	9.494	0.9995	0.1905	0.3501	First order kinetics, Fickian release

### Pharmacological evaluation

Cellulite is characterized by structural changes in the dermis and microcirculation in addition to adipocyte changes, which lead to additional morphological, histochemical, biochemical and ultrastructural modifications. The stimulus for lipogenesis induces adipocyte hypertrophy, which, when accompanied by the formation of fibrous bands, pulls the skin down and creates the characteristic irregular skin shape and dimpling, resulting in the orange-peel-like appearance of the skin^62–64^. Normally, blood glucose level is regulated by several hormones; adiponectin is one of the hormones produced in adipose tissue increasing glucose utilization and inhibiting hepatic gluconeogenesis. In cases of insulin resistance^65,66^, fatty acid breakdown and obesity^67,68^, adiponectin levels are reduced.

**Figure 9:**
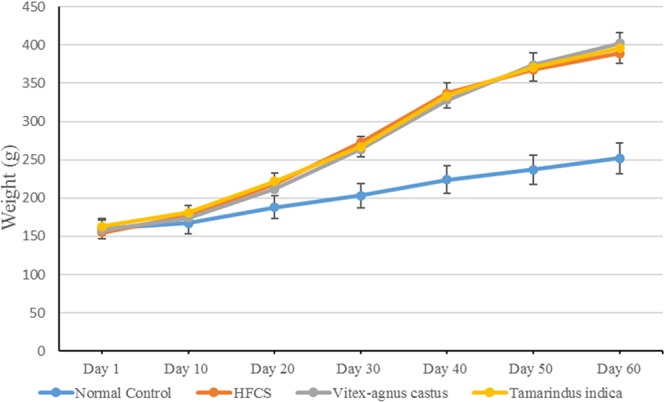
Effect of *V. agnus -castus* and *T. indica* on body weight of HFCS-treated Guinea pig. Body weight was measured and recorded every 10 days.

**Figure 10:**
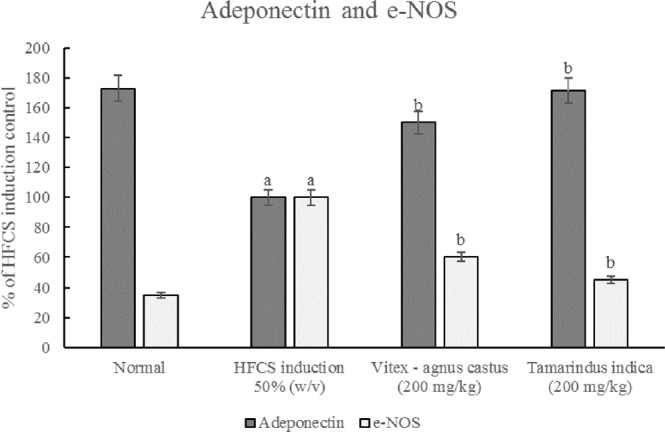
Effect of *V.agnus-castus* and *T.indica* on adiponectin and e-NOS levels in HFCS treated Guinea pigs; ^a^significantly different from normal control group at P ≤ 0.05. ^b^Significantly different from HFCS induction group at P ≤ 0.05.

In this study, body weight gain was induced using HFCS at significantly higher levels (approximately 52%) than those used for normally fed animals (Fig. 9). A hypercaloric diet that is rich in carbohydrates, such as the HFCS diet used in this study, stimulates lipogenesis by increasing lipoprotein lipase activity^63^. Adiponectin levels were significantly reduced in animals fed HFCS, indicating aggravation of the inflammatory condition. This finding was also confirmed by the elevation in serum TNF-α and MPO levels (Table 6). The latter is a protein that is secreted by white blood cells and is a biomarker of inflammation^69^. Additionally, the increase in body weight reported in the current study, triggered the elevation of serum MDA (~63%) along with reduction in reduced GSH (~47%), indicating the presence of oxidative stress (Table 6). Another hallmark of inflammation, is the increase in vascular permeability for which NO has a key role. It has long been believed that a constant amount of NO was produced by eNOS under both physiological and pathological conditions, while the pathological increase in NO was induced by iNOS only. However, this hypothesis has now been rejected due to a lack of sufficient experimental evidence that can completely rule out the involvement of eNOS in these pathological effects; therefore, undoubtedly, eNOS is a much more complex enzyme than presupposed^70^. In the current study, eNOS levels were elevated in animals that received HFCS, as well as under other inflammatory conditions (Fig. 10)^71^, which supports the hypothesis that eNOS might be involved in the pathological effects of NO.

**Table 6:** Effect of *V. agnus-castus* and *T. indica* extracts on TNF – α, MPO activity as well as GSH and MDA tissue content in HFCS-treated guinea pigs.

Groups	TNF-α Pg/g.tissue	MPO U/g.tissue	GSH mg/g.tissue	MDA mol/g.tissue
Normal Control	85.77 ± 3.48	16.49 ± 0.82	306.11 ± 22.12	94.45 ± 7.92
HFCS	145.91 ± 5.93^a^	57.70 ± 2.87^a^	168.47 ± 11.87^a^	152.17 ± 10.17^a^
*V.agnus-castus* (200 mg/kg)	107.99 ± 4.38^b^	42.46 ± 1.72^b^	296.28 ± 18.97^b^	96.53 ± 6.12^b^
*T.indica* (200 mg/kg)	92.79 ± 3.78^b^	24.43 ± 0.99^bc^	355.42 ± 24.14^b^	82.04 ± 5.44^b^

Data are presented as the mean ± SEM; ^a^significantly different from normal control group; P ≤ 0.05; ^b^significantly different from HFCS induction group, P ≤ 0.05; ^c^significantly different from *V. agnus-castus* group at P ≤ 0.05. TNF-α: Pg/g. tissue; MPO: U/g. tissue; GSH mg/g.tissue; MDA: nmol/g.tissue.

**Figure 11:**
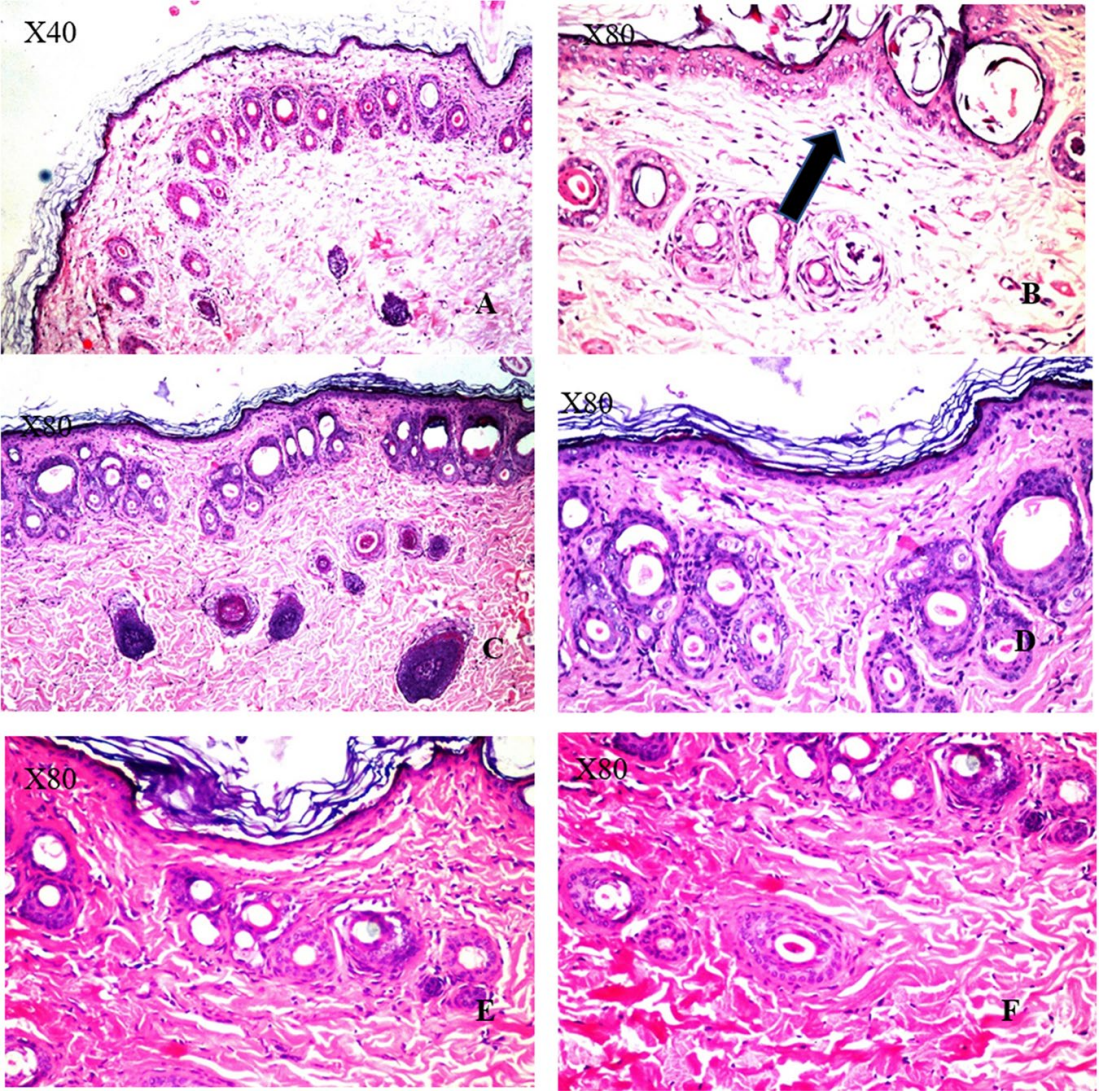
Histopathological structures of skin layers showing changes in the normal structures after intake of HFCS and MNs loaded with *V.agnus-castus* and *T.indica* (200 mg/kg). (**A**) Normal control group with no histopathological alteration and normal epidermis with stratified keratinized epithelium and the underlying areolar connective tissue dermis with hair follicles and glands and lastly the skeletal muscle layer. (**B**) HFCS group: Focal acanthosis was detected in the prickle cell layer of the epidermis (→). The underlying dermis showed oedema, inflammatory cells infiltration and fibroblastic cells proliferation, which were extended deep with appearance of eosinophils infiltration. Necrobiosis was detected in the epithelial cells of some hair follicles. (**C**,**D**) *V. agnus-castus* group: There were hyperkeratosis of the epidermal layer associated with focal hyalinosis in the areolar tissue of the dermis. (**E**,**F**) *T. indica group*: The epidermal and dermal layers were histological intact but there was necrobiosis in some individual hair follicles.

It is well known that the treatment of cellulite using conventional methods is not effective and cannot completely alleviate the symptoms. Therefore, the development of novel therapeutic approaches will aid in the discovery of successful treatments for this distressing condition. In this study, the use of microneedles loaded with *V. agnus-castus* and *T. indica* extracts normalized the oxidative state (MDA, ~33.3% and 46.7%, respectively; GSH, ~86.3% and 118.8%, respectively) and alleviated inflammation (TNF-α ~26%, and 36%, respectively; MPO, ~26.3% and 57.7%, respectively; and adiponectin, ~50% and 70%, respectively). In addition, the MNs loaded with *V. agnus-castus* and *T. indica* extracts restored eNOS levels (40% and 58%, respectively). The pharmacological effects of the MNs loaded with *T. indica* on all the parameters were stronger than those of the MNs loaded with *V. agnus-castus*. However, the effects of the MNs loaded with both extracts were statistically comparable, except for the effect on MPO levels, in which the MNs loaded with *T. indica* produced a significantly greater reduction (~57.7%) than the MNs loaded with *V. agnus-castus* (26.3%). The results were confirmed by histopathological examinations. The untreated group (HFCS) showed oedema, inflammatory cell infiltration and fibroblastic cell proliferation in the underlying dermis and adipocytes that extended to the dermis and epidermal layer compared to the control group (Fig. 11A,B), while treated groups with the MNs loaded with *V. agnus-castus* and *T. indica* showed better skin appearance (Fig. 11C–F).

Despite the beneficial effects of both formulations, neither was able to alter body weight, which supports the idea that body weight or obesity and cellulite are not necessarily directly associated. The pharmacological activity of the MNs loaded with both extracts was greatly attributed to their high phenolic and flavonoid contents in addition to the considerable mineral contents.

## Conclusion

Despite the involvement of inflammation and the well-known alterations in the biochemistry, structure and morphology of the subcutaneous tissue of individuals with cellulite; the pathogenesis of cellulite is yet to be elucidated. In the present work, the authors performed a trial using microneedles, to offer a rapid and painless delivery of drugs (compared to other systemic administration techniques), that ease cellulite manifestations. MNs loaded with *T. indica* extract may help to ameliorate the skin appearance by reducing the inflammatory parameters and improving the antioxidant power. The transdermal delivery of anti-cellulite drugs can be ameliorated through large-scale experimental trials. Ultimately, the use of microneedles offers a simple and relatively cheap way for drug delivery, thus encouraging their wider use in biomedical applications.
